# Personality drives physiological adjustments and is not related to survival

**DOI:** 10.1098/rspb.2013.3135

**Published:** 2014-05-22

**Authors:** Allert I. Bijleveld, Georgina Massourakis, Annemarie van der Marel, Anne Dekinga, Bernard Spaans, Jan A. van Gils, Theunis Piersma

**Affiliations:** 1 Department of Marine Ecology, NIOZ Royal Netherlands Institute for Sea Research, 1790 AB Den Burg, The Netherlands; 2 Animal Ecology Group, Centre for Ecological and Evolutionary Studies, University of Groningen, PO Box 11103, 9700 CC Groningen, The Netherlands

**Keywords:** behavioural syndrome, coping style, phenotypic flexibility, life-history trade-off, pace-of-life, temperament

## Abstract

The evolutionary function and maintenance of variation in animal personality is still under debate. Variation in the size of metabolic organs has recently been suggested to cause and maintain variation in personality. Here, we examine two main underlying notions: (i) that organ sizes vary consistently between individuals and cause consistent behavioural patterns, and (ii) that a more exploratory personality is associated with reduced survival. Exploratory behaviour of captive red knots (*Calidris canutus*, a migrant shorebird) was negatively rather than positively correlated with digestive organ (gizzard) mass, as well as with body mass. In an experiment, we reciprocally reduced and increased individual gizzard masses and found that exploration scores were unaffected. Whether or not these birds were resighted locally over the 19 months after release was negatively correlated with their exploration scores. Moreover, a long-term mark–recapture effort on free-living red knots with known gizzard masses at capture confirmed that local resighting probability (an inverse measure of exploratory behaviour) was correlated with gizzard mass without detrimental effects on survival. We conclude that personality drives physiological adjustments, rather than the other way around*,* and suggest that physiological adjustments mitigate the survival costs of exploratory behaviour. Our results show that we need to reconsider hypotheses explaining personality variation based on organ sizes and differential survival.

## 1. Introduction

Animals modify aspects of their phenotype in response to changes in their environment (phenotypic plasticity [1]). Changes that are reversible within an individual's lifetime are known as phenotypic flexibility [2,3]. Animal behaviour is a classic example of phenotypic flexibility, enabling rapid and reversible responses to changes in environmental and social context [4]. Perhaps somewhat surprisingly, given behavioural flexibility, individuals of many species have been shown to vary consistently in their behaviour across contexts, yielding the notion of ‘animal personalities’ (reviewed in [5]).

Personality refers to a suite of phenotypically or genetically correlated behavioural traits that are consistent over time or across contexts [5–8]. Variation in personality is thought to be shaped by continuous interaction between genes and environment during ontogeny [6,9–13]. In recent years, considerable progress has also been made in understanding personalities from an evolutionary perspective [14–16]. Most of the adaptive explanations involve between-individual variations in state (e.g. physiological condition, health and organ masses), in combination with positive feedback mechanisms maintaining these state variations [14,15,17]. The idea is that if the state of an individual is more or less stable over time, then state-dependent behaviour will also be consistent. However, few empirical studies exist in which predictions from such state-dependent personality models have been tested [17].

The sizes of an individual's metabolic organs (e.g. digestive organs, heart and liver) are thought to be slow-changing state variables that are causal to variation in personality between individuals [18–20]. This variation is thought to be maintained by a positive feedback mechanism, whereby individuals with large metabolic organs behave in ways that allow for the acquisition of enough energy to sustain them. For instance, such individuals might need to be explorative, bold and/or aggressive in order to gain access to the resources necessary for the maintenance of their large organs. At the same time, however, such behaviours are risky and are assumed to come attached with survival costs [20,21]. Exploratory individuals would thus lead a high-risk/high-gain lifestyle. For such behaviour to be evolutionarily stable, the associated survival costs are expected to be compensated for by correlations with particular life-history characteristics (e.g. growth, age at maturity [22]), in line with the ‘pace-of-life’ concept [20]. According to the pace-of-life concept, metabolic costs and personality should be linked along a continuum of slow/fast life-history strategies. However, there is, as yet, little evidence to support this theory [23].

Implicit in the hypothesis that metabolic organ sizes are causal to personality variation is the fact that organ sizes vary consistently between individuals, allowing for consistent behaviour to develop throughout an individual's life. Organs are, however, notoriously flexible in size, reflecting changes in ecological context [2,3,24]. Indeed, regardless of how personalities arise, it seems likely that animals with different personalities will express a preference for different environments (i.e. with respect to food type, predation risk, etc. [15,25,26]), which may, in turn, result in specific physiological adaptations. One could thus argue that personality variation causes consistent variation in organ morphology and, consequently, in metabolic costs, rather than the other way around.

In this study, we examined two critical notions underlying the hypothesis of organ-size-driven personality variation: (i) that variation in digestive organ sizes cause consistent variation in behaviour; and (ii) that large digestive organs and exploratory behaviour are associated with reduced survival. Our model species is the red knot *Calidris canutus* (Linnaeus, 1758), a long-distance migrating shorebird, for which contextual flexibility in organ mass has been extensively studied [3,24,27]. Our study involved four steps. First, we experimentally determined exploratory behaviour for newly captured red knots and correlated this with their digestive organ mass (i.e. the muscular stomach, or gizzard). We also correlated exploratory behaviour with body mass, and predicted that individuals with large body mass (i.e. large energy stores) would avoid risky behaviour and thus be less explorative (*sensu* the mass-dependent predation risk hypothesis [28]). Second, we manipulated gizzard mass in order to compare the effect of a small and a large gizzard on exploratory behaviour within individuals. Third, to test whether the experimental quantification of exploratory behaviour is representative of this behaviour in the field, we tagged and released the experimental birds with unique combinations of colour-rings and estimated local resighting probability. We predicted that explorative birds would have a lower local resighting probability because they have larger spatial ranges than non-explorative birds. Fourth, we analysed survival and resighting probability for free-living red knots, with known gizzard masses, on the basis of a sustained marking and resighting effort on free-living birds [29].

We will show that gizzard mass and body mass (energy stores) were negatively correlated with exploratory behaviour between individuals, and that manipulations of gizzard mass did not cause changes in exploratory behaviour. Moreover, neither gizzard mass nor exploratory behaviour was in any way correlated with survival. We conclude that personality drives the physiological adjustments. These results call for reconsideration of hypotheses explaining personality variation on the basis of organ sizes as well as differential survival.

## 2. Material and methods

### (a) Model species

Red knots are long-distance migratory shorebirds that breed in the High Arctic and spend the rest of the year along more southerly shores with extensive intertidal mudflats [30]. The subspecies *islandica*, studied here, breeds on tundras in northern Greenland and northeast Canada, and winters in northwestern Europe, including the Wadden Sea [30]. During the non-breeding season, red knots roam intertidal mudflats in large flocks in search of burrowed hard-shelled bivalves [31]. Depending on the tides and weather conditions, the availability of the foraging grounds varies temporally and spatially, as does the abundance and quality of prey [32,33].

Bivalves of suitable sizes are swallowed whole and crushed in their muscular stomach, the gizzard [34]. The size of the gizzard sets an upper limit to the amount of shell mass that can be processed and thus limits daily intake rates [35]. Gizzard mass is flexible within individuals and changes in response to the ratio of flesh to shell mass of their prey (prey quality) [36]. The lower this ratio, the larger the gizzard must be to uphold energy intake rates. Gizzard mass is correlated with the mass of other digestive organs such as the intestines, liver and kidneys [27,37]. Together, the digestive organs make up 18% of an individual's lean mass, and are a determining factor for basal and resting metabolic rates [37,38].

Twenty-three red knots were caught between 17 and 20 March 2010 in the Dutch Wadden Sea (53°15′ N, 5°15′ E). A blood sample was taken for molecular sexing [39]. Birds were weighed and ringed on location, whereafter they were transported to the experimental shorebird facility at NIOZ (Texel, The Netherlands; 53°00′12″ N, 4°47′23″ E). Birds were housed in aviaries measuring 4 × 2 m with a height of 2.5 m and lined with white Trespa (Trespa International BV, Weert, The Netherlands). These aviaries provided running saltwater along a coated concrete surface, freshwater for drinking and bathing, and a stretch of sand covered in 5 cm water to resemble the knots’ natural mudflat habitat. The birds were maintained on a diet of protein-rich trout-feed pellets (Produits Trouw, Vervins, France).

### (b) Measuring organ mass

Gizzard mass was measured by A.D. using an ultrasound scanner (model Aquilla, Pie Medical Benelux, Maastricht, The Netherlands) as described by Dekinga *et al.* [36]. Two sets of measurements of gizzard width and height (cm) were taken at each measurement session. Gizzard width and height were averaged per individual, and gizzard mass (g) was derived as −1.09 + 3.78 × width × height (*r* = 0.92, *p* < 0.01; this regression was obtained with fresh gizzard masses from dead individuals). Gizzard mass was measured 1 day after capture (which was taken to be reflective of a birds’ organ mass while free-living), and also 1 day before each treatment of the gizzard mass manipulation experiment.

### (c) Exploratory behaviour

We tested exploratory behaviour in a novel ‘exploration arena’ measuring 7 × 7 m with a height of 3 m (‘novel environment’ test [5]). The exploration arena had walls lined with white Trespa and was filled with a layer of 30 cm seawater on top of a 50 cm deep layer of sand. Filled with only wet sand, we positioned five familiar trays (1 × 1 m, 20 cm deep) above the water surface for the birds to explore. The trays were placed approximately 90 cm from the walls and acted as foraging patches, such that the degree to which birds explored within and between patches would reflect their propensity to explore while searching for food. To further motivate the birds to search for food during the trials, familiar but empty feeders were placed at the centre of each patch. In order to induce standard hunger levels between birds, they were deprived of food for 2 h prior to the experiment, periods without food that knots are accustomed to naturally as they cannot feed around high tide.

Each trial consisted of a bird being retrieved from its aviary, weighed to the nearest 1 g and first introduced into a familiar aviary adjacent to the exploration arena to rest for a minimum of 5 min. This aviary led into the exploration arena through a sliding door that could be remotely opened and closed via a pulley mechanism. After this door was opened, the bird was gently pushed into the exploration arena. Trials lasted 30 min. We tested two to eight randomly selected birds each day between 8 and 11 June 2010, several months after capture. The procedure was repeated between 21 and 24 June 2010.

All trials were recorded on video and later analysed with Observer XT software (v. 10.1, Noldus Information Technology), allowing accurate estimation of time budgets. Our ethogram included ‘searching for food’, ‘resting’, ‘preening’ and ‘flying’. We also scored the patch on which the bird was located at any given time. The logit of the fraction of total time spent in search of food was positively correlated with the log-transformed number of patch visits (*r* = 0.63, *p* < 0.01). Hereafter, we will use the fraction of total time spent in search for food as the measure of exploratory behaviour.

### (d) Separating effects of body mass and energy stores on exploratory behaviour

Many species show a relationship between structural size and body mass. For red knots, however, the principal component from the lengths of wing (mm), tarsus (mm) and head to bill (mm) explained only 16% of variation in body mass within the sexes (electronic supplementary material, appendix S1). In order to investigate correlations between exploratory behaviour and body mass, we analysed these variables in a bivariate mixed-effects model with individual identity as random factor (equations 7a and 7b in [40]). These analyses allowed us to decompose the phenotypic (co)variance and calculate correlation coefficients of exploratory behaviour with body mass between and within individuals. Between-individual and within-individual processes operate in conjunction, and their separation can provide insight into the origin and maintenance of personality variation. Significant correlations between individuals would indicate that behaviour and body mass would take shape by gene–environment interactions during ontogeny, whereas significant within-individual correlations would give hints about more proximate mechanisms. For example, a negative within-individual correlation could indicate that a reduction in body mass (‘hunger’) motivates an individual to explore more. An in-depth discussion on the causes and consequences of between- and within-individual correlations can be found elsewhere [40].

### (e) Gizzard mass treatment

Gizzard mass was manipulated by varying the quality (shell content) of the food [35,36], so that we could measure exploratory behaviour (as described previously) of the same individuals with a large and a small gizzard. To induce a relatively large gizzard, we offered closed blue mussels *Mytilus edulis* that were swallowed whole. To induce small gizzards, we offered only the flesh of the blue mussel, thus removing the need for shell crushing, while keeping the digestible parts identical.

The 23 knots were divided into two groups of 11 and 12 individuals, respectively. One group started with the large gizzard mass treatment followed by the small gizzard mass treatment, while the other group was simultaneously exposed to the two treatments in reversed order (a crossover design to avoid confounding effects of time). In captivity, it takes about a week for a bird's gizzard mass to match its diet [36]. We allowed at least three weeks for the birds to increase gizzard mass after a diet switch. Trials were conducted between 21 December 2010 and 21 January 2011, after which the birds were returned to a diet of trout-feed pellets.

In order to account for variation in magnitude of gizzard mass change, as well as to decompose the (co)variance into the between- and within-individual components, we analysed exploratory behaviour and gizzard mass in a bivariate mixed-effects model with individual identity as a random effect [40]. We did not include the initial gizzard mass measurements in this analysis, as there was no corresponding measure of exploratory behaviour at that time. The effect of the order in which birds received the gizzard manipulation was not significant (−0.19, 95% CI (−1.23; 0.77)), and for simplification we removed it from the final model. In order to test whether individuals varied consistently in gizzard mass between treatments, we calculated ‘consistency repeatability’ from standardized gizzard mass [41].

### (f) Free-living exploratory behaviour of experimental birds

In August 2011, after the experiments had been completed, all birds (except for two that had died) were released into the wild (53°15′ N, 5°15′ E). A week before their release, the birds were fed blue mussels and tagged with unique colour-coded ring combinations placed around their legs allowing for individual identification in the wild [29]. Resightings of these individuals up to March 2013 allowed us to estimate their free-living exploratory space use.

### (g) Long-term resighting analyses of free-living birds

Between 1998 and 2003, 402 *islandica* knots were captured and promptly released in the Dutch Wadden Sea after their gizzard mass had been measured, and they had been tagged with unique colour-coded combinations of rings. Resightings of these birds in the Dutch Wadden Sea (*n* = 1068) were analysed over the period from capture up to March 2013 to estimate ‘apparent survival’ and resighting probability. Note that apparent survival includes true survival as well as permanent emigration, which cannot be separated [42]. In order to correct for food-type- and season-induced variation in gizzard mass between and within years [35], we zero-centred gizzard mass for each catching event (*n* = 16) [43].

### (h) Data analyses

For each captive individual, exploratory behaviour was measured on four occasions: two replicates during the first quantification of exploratory behaviour, and two replicates during the gizzard mass manipulation. Fraction of time spent searching in the exploration arena (exploratory behaviour) was logit-transformed to conform to normality assumptions. Repeatability *R* in exploratory behaviour was calculated as the between-individual variance divided by the total phenotypic variance; that is, the sum of between- and within-individual (residual) variance. Variance components were extracted from a univariate mixed-effects model with individual identity as a random effect. Confidence intervals and significance were calculated with parametric bootstrapping [41]. We initially included sex as a fixed effect, but we removed this from the final model as exploratory behaviour did not significantly differ between males and females (0.3 s.e. 0.6). In order to truly capture the effect of a ‘novel’ environment, we correlated gizzard mass at capture to exploratory behaviour from the first replicate. Because our purpose was not to predict exploratory behaviour from gizzard mass, but only to summarize their relationship, we used standardized major axis analyses [44].

Apparent survival and resighting probabilities were calculated from resighting histories of free-living individuals using the statistical software MARK [42]. Our candidate model set included models with fixed or residual gizzard-mass-dependent apparent survival and resighting probability. To account for variation in apparent survival and resighting probability between years, we additionally included models with time-dependent apparent survival and resighting probability in our candidate model set (i.e. year as factor with 15 levels). For model selection and inference, we used Akaike's information criterion corrected for small sample size (AICc). In order to test for violations of the assumptions underlying mark–recapture analyses, we performed a goodness-of-fit test of the global model without covariates, including time effects on apparent survival and resighting probability, using the program U-CARE [45]. These results indicated that our model fitted the data adequately (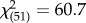, *p* = 0.17).

Data analyses were carried out in R v. 2.15.1 [46] with the packages ‘RMark’ for mark–recapture [47], ‘rptR’ for univariate mixed-effects repeatability [41], ‘smatr’ for standardized major axis [48] and ‘MCMCglmm’ for bivariate mixed-effects analyses [49].

## 3. Results

### (a) Exploratory behaviour

**Figure 1.**
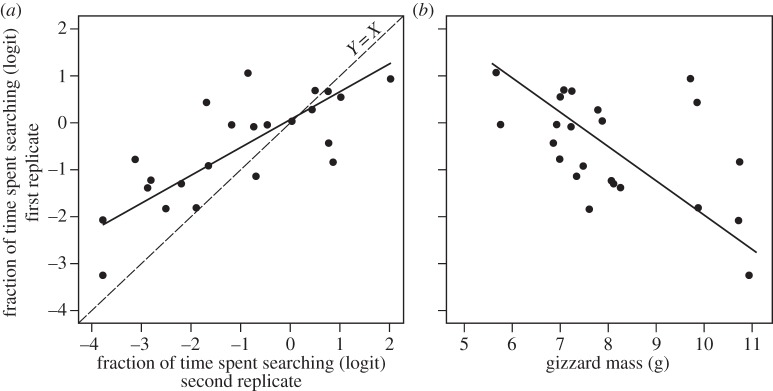
Standardized major axis regressions (*a*) between the first measure of exploratory behaviour and the second, as well as (*b*) gizzard mass. Gizzard mass was measured shortly after capture, and is therefore representative for this organ mass in the wild. Exploratory behaviour was measured as the fraction of time spent searching and was logit-transformed.

**Figure 2.**
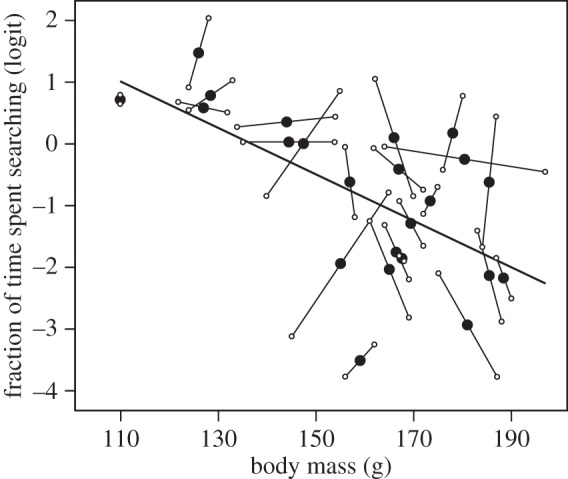
Between- and within-individual correlations of body mass with exploratory behaviour. The closed circles represent an individual's average body mass and exploratory behaviour of the first two exploration trials. The line represents the between-individual standardized major axis regression as estimated from the bivariate mixed-effects model. The open circles depict an individual's body mass and exploratory behaviour at each trial. Exploratory behaviour was measured as the fraction of time spent searching and was logit-transformed.

Our first set of experiments revealed that exploratory behaviour was repeatable (*R* = 0.67, 95% CI (0.38; 0.85), *p* < 0.01; figure 1*a*), and that it was negatively correlated with gizzard mass at capture (intercept = 5.3, 95% CI (3.0; 7.6), slope = −0.72, 95% CI (−1.06; −0.50), *r* = −0.52, *p* = 0.01; figure 1*b*). Within individuals, a reduction in body mass (energy stores) did not motivate birds to explore more, as the within-individual correlation of exploratory behaviour with body mass was non-significant (*r* = 0.13, 95% CI (−0.35; 0.44); figure 2). There was, however, a significant and negative between-individual correlation of exploratory behaviour with body mass (*r* = −0.84, 95% CI (−0.96; −0.45); figure 2). Body mass during these trials was correlated with body mass at capture, indicating that body mass in captivity reflects body mass while living free (*r* = 0.59, 95% CI (0.24; 0.81), *t*_21_ = 3.4, *p* < 0.01).

### (b) Gizzard mass treatment

**Figure 3.**
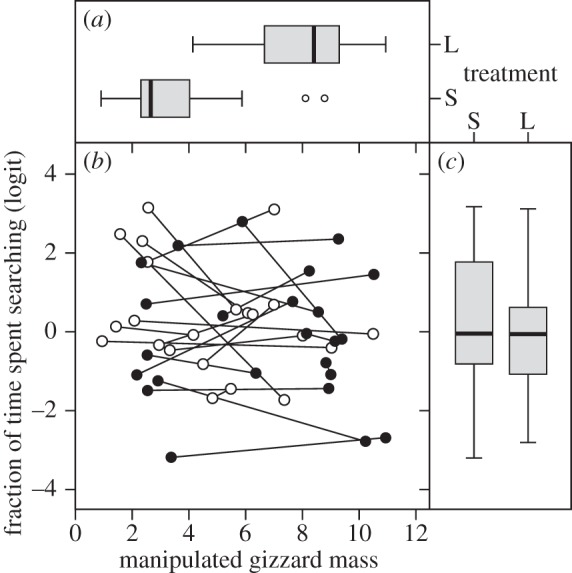
Gizzard mass and exploratory behaviour as a result of the gizzard mass manipulation. Different panels represent (*a*) gizzard mass as function of treatment level, (*b*) exploratory behaviour as a function of manipulated gizzard mass and (*c*) exploratory behaviour as a function of treatment level. Treatment level refers to the small gizzard (S) or large gizzard treatment (L). Exploratory behaviour was measured as the fraction of time spent searching and was logit-transformed. We analysed the effect of the gizzard mass treatment on exploratory behaviour in a bivariate mixed-effects model to accommodate the differences in magnitude of gizzard mass change between individuals. Lines connect individuals between treatment levels. Closed circles in panel (*b*) represent individuals that first received the large and then the small gizzard mass treatment; open circles represent individuals that were simultaneously given the reverse treatment order.

Manipulating gizzard mass resulted in an average gizzard mass difference of 4.6 g between treatments (s.e. 0.6, ANOVA: *F*_1,44_ = 66.7, *p* < 0.01; figure 3*a*). An individual's exploratory behaviour did not change in response to the manipulation of gizzard mass, as evidenced by a lack of within-individual correlation of exploratory behaviour with manipulated gizzard mass (*r* = −0.20, 95% CI (−0.50; 0.11); figure 3*b*). Between individuals, the correlation of exploratory behaviour with manipulated gizzard mass did not differ significantly from zero either (*r* = −0.40, 95% CI (−0.90; 0.69); figure 3*c*). The absence of this correlation compared with the negative between-individual correlation we found when the birds were living free suggests that gizzard mass is not determined by individual ‘design’ constraints (e.g. genetic architecture and body size), but regulated by diet. Indeed, manipulated gizzard mass (when diet was controlled for) was not repeatable (*R*_consistency_ = 0.22, 95% CI (0.00; 0.55), *p* = 1.00). By contrast, exploratory behaviour in the gizzard manipulation trials was repeatable (*R* = 0.56, 95% CI (0.22; 0.79)), also with respect to the first measure of exploratory behaviour six months before (*R* = 0.54, 95% CI (0.21; 0.77), *p* < 0.01). Surprisingly, however, exploratory behaviour was no longer significantly correlated with gizzard mass at capture. Nonetheless, the estimated values for intercept (7.3) and slope (−0.89) were within the 95% confidence intervals ((3.9; 10.6), *p* = 0.24; and (−1.38; −0.57), *p* = 0.34, respectively) of those estimated from the correlation between the first measures of exploratory behaviour and gizzard mass at capture.

### (c) Free-living exploratory behaviour of experimental birds

**Figure 4.**
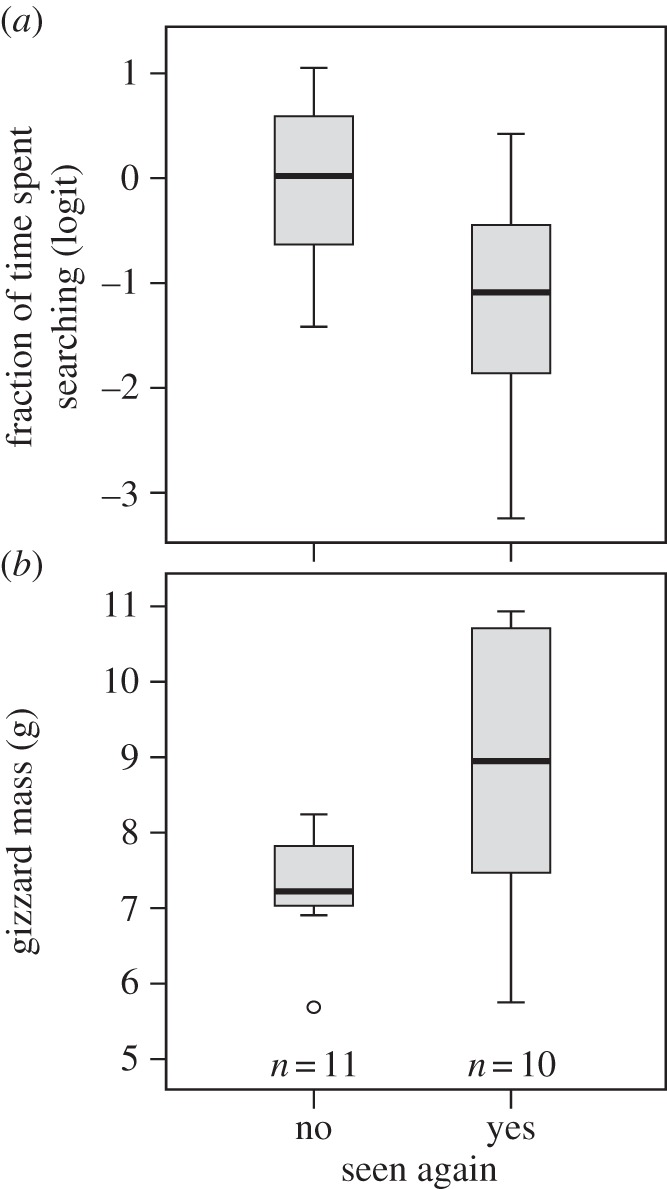
(*a*) Exploratory behaviour and (*b*) gizzard mass for red knots that were, and were not, resighted in the Dutch Wadden Sea in the period between being released (August 2011) and March 2013. Exploratory behaviour was measured as the fraction of time spent searching in the first measurement of exploration and was logit-transformed.

Out of 21 experimental birds that were released in the wild, 10 were resighted in the period between release and March 2013. In line with our experimental results, free-living exploratory individuals with small gizzards had a lower resighting probability than non-exploratory individuals with large gizzards. Birds that were not resighted had significantly higher exploratory behaviour scores (1.1 s.e. 0.4, ANOVA: *F*_1,19_ = 7.2, *p* = 0.01; figure 4*a*) and smaller gizzard masses (−1.5 s.e. 0.6, ANOVA: *F*_1,19_ = 6.0, *p* = 0.02; figure 4*b*) than birds that were resighted.

### (d) Long-term resighting analyses of free-living birds

**Figure 5.**
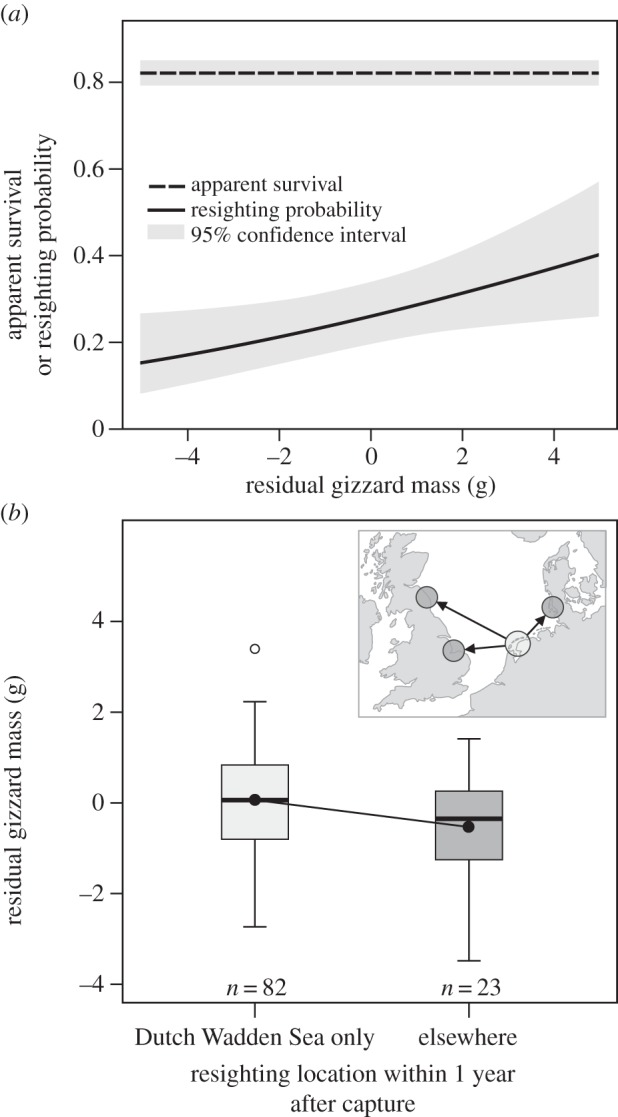
Resighting analysis of free-living red knots with known gizzard mass. (*a*) Apparent survival and resighting probability in the Dutch Wadden Sea as a function of residual gizzard mass at capture, and (*b*) average residual gizzard mass for birds resighted in the Dutch Wadden Sea only and those elsewhere within 1 year after capture. Those knots that were resighted outside the Dutch Wadden Sea within a year after capture were resighted in England or Germany (see inset of panel (*b*)).

Based on the analysis of our long-term resighting efforts and in line with our independent experimental results, we found that exploratory behaviour and gizzard mass were negatively correlated in the field as well. The logit of resighting probability increased by 0.13 (95% CI (0.02; 0.24)) per gram of residual gizzard mass (figure 5*a*; electronic supplementary material, tables S1 and S2), meaning birds with small gizzards were less often resighted in the Dutch Wadden Sea than those with large gizzards. Similarly, the average gizzard mass of individuals that were resighted outside the Dutch Wadden Sea within a year after capture was lower than that of individuals that were resighted in the Dutch Wadden Sea only (−0.80 s.e. 0.37, *F*_1,108_ = 4.7, *p* = 0.03, figure 5*b*). We did not find an effect of gizzard mass on apparent survival, which averaged 0.82 (95% CI (0.79; 0.84); electronic supplementary material, tables S1 and S2), suggesting that neither large metabolic machinery nor exploratory behaviour are associated with lower survival.

## 4. Discussion

Consistent variation in (metabolic) organ mass has been hypothesized to cause variation in personality traits [18–20]. In this study, we examined two critical notions underlying this hypothesis. Instead of the hypothesized *positive* between-individual correlation, we found that exploratory behaviour was *negatively* correlated with digestive organ (gizzard) mass. To examine the causality of this correlation, we manipulated gizzard mass and found that an individual's exploratory behaviour was unaffected. This led us to reject the hypothesis that variation in digestive organ size causes consistent exploratory behaviour within individuals. For free-living knots, we also showed that exploratory behaviour was negatively correlated with gizzard mass between individuals, and that neither factor was associated with lower survival. Consistent variation in exploratory behaviour, or some correlated variable, seems to cause variation in digestive organ mass.

### (a) An ecology of exploratory behaviour

Consistent differences in exploratory behaviour are found in many different organisms [5]. Usually, exploratory behaviour is measured in standardized experiments outside an individual's regular environment, which can be problematic for the interpretation of the trait under investigation [50]. To avoid ambiguity in the measurement of personality traits, validation against behaviour in the wild is essential [5,50]. Nonetheless, a few studies show that small-scale exploratory behaviour in a laboratory is related to large-scale space use in the wild. In one example, after removal of a food source, non-explorative great tits *Parus major* remained close to the known feeder location, whereas explorative individuals moved further away [51]. Comparable results were found for brook charr *Salvelinus fontinalis* [52], starlings *Sturnus vulgaris* [53] and red squirrels *Tamiasciurus hudsonicus* [21]. For red knots, we now show that exploratory behaviour in a laboratory setting is also related to space use in the wild on a spatial scale of northwestern Europe, which is unprecedented. The experimental birds that were not resighted in the local study area after release had higher experimental exploration scores than birds that were locally resighted. The explorative individuals with small gizzards spread out on spatial scales of up to hundreds of kilometres between mudflats in England, The Netherlands and Germany.

An individual's gizzard mass is flexible and reflects the quality of prey that it consumed over the previous few weeks [36]. Experimental exploration scores were negatively correlated with gizzard mass in the wild, suggesting that exploratory behaviour is correlated with prey type between individuals, either directly or indirectly (e.g. through increased access to areas where high-quality prey are available). Furthermore, the positive between-individual correlation of resighting probability with residual gizzard mass at capture was present in all years (1998–2013) after capture (between 1998 and 2003). The temporal consistency of this correlation suggests that an individual's exploratory behaviour is consistent over time and that gizzard mass is indeed behaviourally regulated.

One could argue that the between-individual correlation of exploratory behaviour with gizzard mass has been formed by the interaction between genetic mechanisms (e.g. coevolution, pleiotropy and linkage disequilibrium) and environmental mechanisms (e.g. permanent environmental correlations) [40]. The lack of repeatability in an individual's gizzard mass between the small and large gizzard mass treatment, however, does not support such an argument. Gizzard mass might still be regulated by an underlying unknown process (e.g. prey preference) that itself is correlated with exploratory behaviour. A particularly interesting mechanism that, in theory, could be capable of generating the observed correlation between exploratory behaviour and gizzard mass during ontogeny [14,17] is a positive feedback mechanism between gizzard mass and prey quality.

In the Wadden Sea, prey quality is inversely related to prey density [33], and the spatial extent where high-quality prey are available is limited [32]. Because of a digestive constraint, individuals with small gizzards can only achieve sufficiently high intake rates on a diet of high-quality prey (i.e. there is a positive feedback between gizzard mass and prey type) [33–35]. As high-quality prey is less abundant than low-quality prey, it was previously thought that birds with small gizzards would have an increased starvation danger compared with birds with large gizzards [54]. We have now shown that there is no survival cost for having a small gizzard, which is at odds with this notion. Possibly, the increased starvation risk of having a small gizzard can be compensated for by being explorative, thereby allowing the discovery of high-quality prey.

### (b) Exploratory behaviour, survival and body mass

The evolutionary origin and maintenance of phenotypic variation in animal personality is intensely debated [15,17,55,56]. Recent work suggests that personality variation among individuals could reflect variation in adaptive specialization to a particular life-history strategy (the ‘pace-of-life’ concept [20]). Explorative individuals are likely to incur costs associated with movement that may reduce survival (e.g. through increased metabolic costs and higher predation danger through increased exposure [21,27]). From a life-history strategy perspective, these survival costs are expected to be compensated for by increased growth, age at maturity and reproduction if exploratory behaviour is to be evolutionarily stable (a high-risk/high-gain lifestyle [20,22]). Empirical evidence that there are survival costs to exploratory behaviour, however, is equivocal [57]. Our results do not provide any evidence that exploratory behaviour is associated with reduced survival.

**Figure 6.**
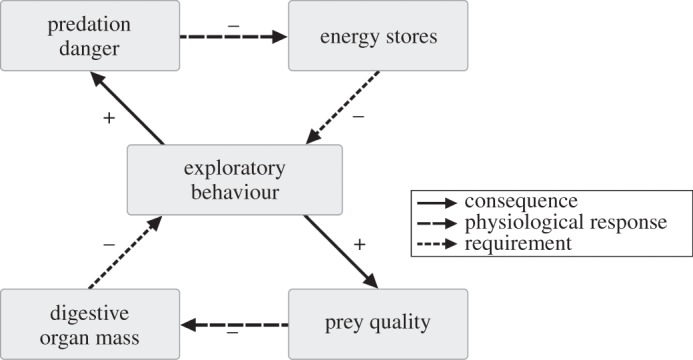
Hypothesized positive feedback loop capable of maintaining variation in exploratory behaviour between red knots. The consequence of exploratory behaviour is increased predation danger, to which red knots respond physiologically by having lower energy stores. Low energy stores increase starvation danger, which requires exploratory behaviour that consequently enables the discovery of high-quality prey. Digestive organ mass will be small as a physiological response to consuming high-quality prey, which in turn requires exploratory behaviour enabling the discovery of sparsely distributed high-quality prey, because birds with small gizzards can only achieve a sufficient intake rate on high-quality prey.

Other than through adaptive specialization to a particular life-history strategy, costs of an individual's personality could be reduced through correlations with other traits such as body mass [4,12]. For instance, exploratory blackbirds *Turdus merula* compensated for increased flight costs and predation danger by carrying smaller energy stores than more sedentary individuals, albeit at the cost of increased starvation danger [58]. Similarly, we found a negative correlation of body mass with exploratory behaviour between individuals. Red knots show relatively small variations in structural size [59], and the observed mass differences between exploratory and non-exploratory birds (maximum of 79 g) are too large to be accounted for by differences in organ mass only [37]. Moreover, in an experimental setting, red knots have been shown to actively reduce body mass in the presence of predators [60], allowing better escape behaviour from predators [61]. Birds with small energy stores could compensate for their increased risk of starvation by searching for higher-quality prey. Indeed, in our study, lighter birds were more explorative. This effectively creates two positive feedback loops (figure 6): one between exploratory behaviour, predation danger and energy stores, and another between exploratory behaviour, prey quality and digestive organ mass. Although in our short-term laboratory study with fully mature birds we did not find a within-individual correlation between exploratory behaviour and gizzard mass, nor body mass, in the field the situation is expected to be different for two reasons. First, in the more demanding lifestyle of the wild, exploration for the sparsely distributed high-quality prey is required for individuals with small gizzards and energy stores, which are digestively constrained [33–35]. Likewise, having small energy stores will increase the risk of starvation, and thus require birds to be more explorative and increase the probability of finding (high-quality) prey. Second, we imagine that such feedback loops are especially important during ontogeny (either or not in interaction with genetic dispositions), after which behaviour could become fixed to some extent (i.e. consistent). Small differences in any of the variables in the hypothesized feedback loops could lead to lasting between-individual differences. For example, if, by chance, a young and learning individual experiences an unsuccessful foraging bout, and consequently low energy stores, it will be prompted to explore more, facing higher predation risk, and thus enforcing maintenance of lower energy stores [28]. At the same time, exploratory behaviour allows access to high-quality prey, wherefore birds will acquire small gizzards, thus enforcing exploratory behaviour (figure 6). The challenge is to pinpoint whether, and at what time during ontogeny, consistent variation in behaviour and physiology will start to occur. For such investigations, we need to understand the key state variables involved in the trajectory towards exploratory or non-exploratory personalities. We propose that the causal framework sketched in figure 6 could be the working hypothesis upon which to build further empirical and theoretical work.

## Notes

All experiments were carried out under DEC protocol NIOZ 10.01 and according to Dutch law.

## Data accessibility

The data underlying this study are made available on Dryad: doi:10.5061/dryad.jr934 [62].
